# Research protocol: investigating the feasibility of a group self-management intervention for stroke (the GUSTO study)

**DOI:** 10.1186/s40814-017-0220-1

**Published:** 2018-01-11

**Authors:** Ella Clark, Nick S. Ward, Gianluca Baio, Fiona Jones

**Affiliations:** 1 National Hospital for Neurology and Neuroscience, University College London Hospital, Box 146, 33 Queen Square, London, WC1N 3BG England; 2 Sobell Department of Motor Neuroscience and Motor Disorders, University College London, London, England; 3 Department of Statistical Science, University College London, London, England; 4 Faculty of Health, Social Care and Education, Kingston University and St Georges University of London, London, England

**Keywords:** Stroke, Self-management, Self-efficacy, Group programmes, Rehabilitation, Long-term conditions, Feasibility

## Abstract

**Background:**

Life after stroke can be an ongoing struggle with over half of all survivors reporting unmet emotional and social needs. In the United Kingdom’s (UK) national clinical guidelines for stroke, self-management is suggested as one approach which can support long-term needs. In the UK NHS, self-management interventions are delivered in various ways. Regardless of the delivery mechanism, a tailored approach and ways to integrate peer support are advocated. Group delivery offers a platform for peer support and has the potential to remain individualised. However, before the efficacy of a group self-management intervention can be tested, the feasibility must be explored. This research investigates the feasibility of a GroUp Self-management intervention for sTrOke (GUSTO).

**Methods:**

A randomised waitlist control design will be used to investigate the feasibility of a group self-management intervention adapted from an existing one-to-one intervention called *Bridges*. A mixed methods approach will be used. Qualitative work will capture participant experience, while quantitative work will allow preliminary comparison between the intervention and waitlist groups (between subjects) and pre-post intervention measures (within subjects). Interviews will be conducted with stroke survivors and focus groups with family and friends to assess acceptability of the intervention.

**Discussion:**

There is a growing interest in group-based self-management interventions for stroke as a method of supporting stroke survivors’ ongoing unmet needs. This is an area with limited research to date. This study will inform design of a fully powered trial which would assess the efficacy of a group self-management intervention following stroke.

**Trial registration:**

ISRCTN19867168

**Electronic supplementary material:**

The online version of this article (10.1186/s40814-017-0220-1) contains supplementary material, which is available to authorized users.

## Background

At present, there are 1.2 million people living with the effects of stroke in the UK [1]. These effects are varied, but over three quarters of survivors report limb weakness, half report unmet needs relating to social and emotional issues and a third report a communication disorder [1, 2]. The long-term nature of these effects has led to suggestions that stroke should be defined as a chronic disease albeit one that starts with an acute event [3]. Although there have been improvements in acute care services, once care ends many stroke survivors feel ‘abandoned’ [4] and perceive living with stroke as an ‘ongoing struggle’ [5]. The ‘alarming neglect’ in the development of services available to survivors in the long term has been noted, and there is a call for better long-term support [3, 6].

In order to improve support for individuals living with chronic diseases such as stroke, health care professionals need to ‘abandon’ traditional ways of thinking [6]. Current services often place high expectation on the individual to manage and take responsibility for their condition [4, 7], which can result in significant variation in the extent to which survivors are supported to manage life after stroke [8]. New approaches are needed, for example, one way of supporting survivors increasingly recognised by national policy is a tailored self-management approach [9]. Broadly speaking, self-management supports individuals ‘to manage their health on a day-to-day basis’ [10], through increasing an individual’s belief in their own ability to engage with specific tasks [11].

Self-management interventions specifically for stroke are relatively new. It is thought they have emerged after the success of interventions such as the Stanford Chronic Disease Self-Management Programme which has been implemented for a range of chronic conditions [12]. Although chronic conditions vary greatly in presentation, there are a number of similarities in the way they are managed which suggests similar strategies could be applied to stroke [13]. A recent Cochrane review explored self-management interventions specifically for stroke and found significant effects in favour of the self-management interventions for both quality of life and self-efficacy [14]. Despite their variability in the delivery of the interventions, those based on the concept of self-efficacy showed most promise.

One example of a self-management intervention, based on self-efficacy and currently integrated into some stroke teams within the UK’s National Health Service, is *Bridges self-management or Bridges* [15]. Bridges is underpinned by self-efficacy principles and offers a unified way of working for stroke teams which enables flexibility through context adaption such as the setting or time post stroke, ensuring the content remains tailored and personalised. The intervention is currently delivered on a one-to-one basis; however, the notion that self-management is an ‘individual construct’ is questioned with some suggesting self-management is a ‘collective process where social networks can potentially make a considerable contribution to improving health outcomes’ [16]. Evidence for this comes from stroke survivors themselves who report the degree to which their self-manage is influenced by the social context they operate within [17].

Interviews with 37 stroke survivors who had previously completed a self-management intervention delivered in a group setting offered insight into the numerous benefits of peer support [18]. These included normalisation of one’s situation through the sharing of experiences, a source of motivation, and the opportunity to learn from others. To explore this further, 14 stroke survivors with no experience of group self-management interventions were interviewed. Although the concept of group self-management interventions was acceptable to stroke survivors, and seen to have potential benefits, ensuring the intervention remained tailored was an area of concern. To advance the field of stroke self-management, an intervention based on self-efficacy, which enables peer support and has the flexibility to remain tailored, is required.

Bridges meets two out of three of these criteria, but one-to-one delivery does not maximise the opportunity for peer support. Thus, there is interest in whether it could be adapted to a group setting. Further development of Bridges has been encouraged and in a recent paper was conceptualised as a ‘boundary object’, emphasising that it is constantly evolving in nature [10]. However, it is currently unknown whether Bridges would be feasible or effective if delivered in a group setting. Prior to examining efficacy, it is important to establish whether Bridges *could* be adapted for this purpose. This type of work is defined by National Institute for Health Research (NIHR) as feasibility [19] and provides parameters essential for future work.

This paper will present the protocol for research designed to address the question: is it feasible to deliver Bridges self-management as a group intervention?

### Sub questions

In order to address whether it is feasible to deliver the Bridges self-management intervention in a group setting, the following specific sub questions will be addressed:

1. How many stroke survivors are eligible for the group self-management intervention and how quickly can they be recruited?
2. Are participants willing to be randomised to a waitlist control in a group self-management study?
3. What will the level of adherence and attendance to the group self-management intervention be?
4. What will follow-up response rates to questionnaires be?
5. How acceptable to stroke survivors is it to deliver Bridges in a group setting?
6. How acceptable is it to family/friends/carers to deliver Bridges in a group setting?
7. What is the magnitude and variability of change in outcome measures post intervention?

## Methods

The Standard Protocol Items: Recommendations for Interventional Trials (SPIRIT) checklist was used to ensure relevant data was reported in this protocol [20]. In addition, relevant guidelines were followed including the Medical Research Council (MRC) guidelines for complex interventions [21], Template for Intervention Description and Replication (TiDieR) guidelines for intervention descriptions [22], SPIRIT reporting guidelines [20] and NIHR advice on feasibility studies [19]. This is the first published version of the study protocol (11.04.2017).

### Approvals

Ethical approval has been obtained for this study (see the ‘Ethics approval and consent to participate’ section). The study is registered on the ISRCTN registry (ISRCTN19867168) on 20.12.2016. This study is funded by NIHR who peer reviewed the study (see the ‘Funding’ section).

### Study design

The study is designed in accordance with the guidelines listed above to answer the question: is it feasible to deliver the Bridges self-management intervention in a group setting? As recommended by guidelines, the study will use a mixed method approach [23]. A randomised waitlist control design will be used and stroke participants will be recruited to the intervention (see Table 1) at different time points. This design will allow comparison between the intervention and waitlist groups (between subjects) and pre-post intervention measures (within subjects). The former will allow sample size calculations to be made. Outcome measures (described in more detail below) collected post intervention also allow any long-term effects of the intervention to be assessed. Once the intervention has been delivered, interviews will be conducted with a sample of the research participants and focus groups will be held with family and friends. Any changes to the protocol are to be discussed with the funders, ethics committee and management panel. A SPIRIT diagram illustrating the course of the study is shown in Table 1.

**Table 1:**
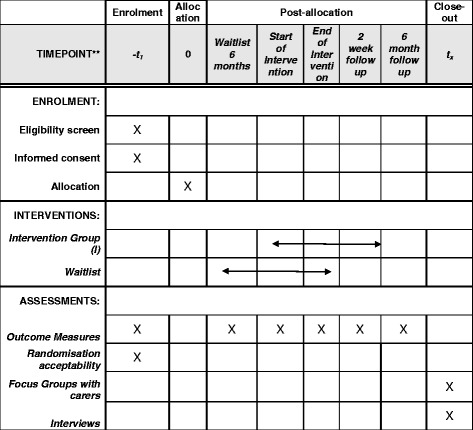
Standard Protocol Items: Recommendations for Interventional Trials (SPIRIT) diagram illustrating study design

### Recruitment

#### Sample size

The National Institute for Health Research guidelines state no formal power calculation is needed for feasibility studies such as this one [19]. Our sample size is based on published guidance for feasibility studies which varies in recommendation from 24 to 50 participants [24, 25]. We have based our sample size on the most conservative estimate for group size (*N* ≥ 50). Adherence to Bridges on a one-to-one level has been reported at 100% [26]. Dropout rates from group Bridges may be different, but this is currently unknown. A conservative estimate of 10 dropouts gave a total sample size of 60 participants.

#### Stroke survivors

Recruitment will be overseen by a Clinical Research Network. The setting will be a Hyper Acute Stroke Unit (HASU) in a central London Hospital. The intense nature of HASU means patients are initially consented to a Permission To Contact register (PTC) and given information about the study, allowing individuals to read the information sheet in their own time. The PTC is a list of those who have given permission to be contacted by researchers in the future. It enables access to a naturally occurring sample within the stroke population which minimises the risk of selection bias and maximises the chance the sample is representative of the target population. The PTC has been recruiting stroke survivors since 2013. As a result, it contains individuals who had their stroke up to 2 years previously, offering a greater range of time post stroke than if recruiting from the start of this research. A limitation of the PTC used for the proposed study is that it only comprises patients who can consent for themselves during their stay in HASU so may result in a sample of individuals with less severe strokes. This will be acknowledged in the final analysis and write up of findings.

After December 2015, the PTC was adapted to become the PTC+ which does include patients that are unable to consent for themselves (a family of friend can consent on their behalf). This could minimise a possible selection bias towards participants with less severe strokes. To gain the benefits of both these registers, we will recruit from the PTC for the first 15 participants and the PTC+ for the remaining 45. Recruitment will start from the first entry on each register and continued in chronological order. A member of the research team will enrol participants in the study (including consent). Once an individual has been enrolled by a member of the research team, they will be randomly allocated to a condition by a statistician using blocked randomisation to ensure balanced numbers. The first patient was enrolled on 25/02/2016. Question 1 (how many stroke survivors are eligible for the group self-management intervention and how quickly can they be recruited?) will be answered through documentation of this recruitment process.

#### Inclusion and exclusion criteria

As this work is feasibility, the exclusion criteria are kept broad to gain insight into who the group self-management intervention may be appropriate for. For example, it is unknown whether the group self-management intervention would be accessible for people with aphasia so this was not an exclusion criterion.

- *Inclusion criteria* are as follows:
  - Confirmed diagnosis of stroke from medical professional
  - Able to understand a two-stage command at time of consent either verbally or non-verbally
  - Able to understand English (no interpreter is available but participants would be encouraged to come with a family or friend supporter who could assist with interpretation)
  - At least 18 years old
  - Discharged from community rehabilitation services
- *Exclusion criteria* are as follows:
  - Any previous access or support from Bridges self-management intervention
  - Severe aphasia defined as being unable to follow a two-step command
  - Clinical diagnosis of depression from medical professional
  - Severe co-morbidities such as malignancy or unstable cardiovascular condition
  - Unable to hold a conversation in English and no supporter/friend available to translate

#### Randomisation

Participants will be randomised by a member of the research team using a blocked randomisation chart created by the statistician on the management panel (GB). Blocked randomisation was chosen as it ensured balanced numbers were allocated to the intervention and the control group, something pure randomisation cannot provide [36]. The randomisation process was unblinded, as researchers knew which condition the participants would be allocated to.

#### Family, friends and carers

Question 6 (how acceptable is it to family/friends/carers to deliver Bridges in a group setting?) will be answered by inviting family/friends/carers who accompanied a stroke survivor to the group self-management intervention to take part in a focus group. Recruitment will be determined by purposeful sampling to ensure a range of demographics such as age, gender and ethnicity, which group the individual attended, whether they had aphasia and time post stroke. Every carer who attends the group will be invited to take part in a focus group. We will aim for a minimum of four and maximum of 12 carers for each focus group.

### Intervention group

Individuals randomised to the intervention group by a statistician will complete the intervention as soon as they are enrolled. The intervention consists of four consecutive sessions over 4 weeks. They will complete baseline measures before the intervention starts and prior to randomisation. Demographic information will be gathered at baseline for all participants which included age, gender, date of stroke, co-morbidity, aphasia, living situation, ethnicity and employment status. Once they finish the intervention, they will complete outcome measures at three time points: at the end of intervention and then at 2 weeks and 6 months post intervention. Participants may withdraw from the intervention group at any time if they request to do so.

The intervention is a group self-management intervention for stroke which was adapted from the existing one-to-one Bridges self-management intervention. It was designed over a 12-month period (February 2015–February 2016). Five sources were consulted to facilitate the adaption process as follows:

- MRC guidelines for complex interventions [23]
- A systematic review of current group self-management interventions (currently unpublished) explored the key features and role of peer support which in turn informed content and structure
- The existing Bridges self-management intervention [15] provided core principles and insight to potential challenges that may present during the delivery of the intervention. Also, a Bridges workbook was given to all participants (see Table 1).
- Interviews with stroke survivors about group self-management interventions [27] provided insights including the need for a group intervention to remain tailored, be in an accessible venue and suggested the addition of a stroke survivor joint-facilitator
- A focus group with stroke survivors exploring desirable content. The focus group was made up of seven stroke survivors and one young carer and was facilitated by FJ, the founder of Bridges. Insights such as how to present the group and the need for a separate time for socialising were given.

The resulting group self-management intervention is described in Table 2 using headings suggested by guidelines [22].

**Table 2:** Intervention reporting in line with the Template for Intervention Description and Replication (TiDieR) guidelines

Name	Group self-management intervention for stroke
Why (rationale)	Described above in the ‘Background’ section.
What (materials)	Stroke survivors will receive a user held Bridges workbook which they can take away with them and work through at their own pace. The layout and content of the workbook has vignettes, pictures and experiences from other stroke survivors. It was designed with stroke survivors and has been reviewed by Connect (communications charity). Family and friends will receive a Bridges family and friends booklet containing information about SM, stories from families living with stroke, the five top tips for supporting target setting and finally, resources and contacts. Both are available through Bridges [15]. In addition, flip chart paper, post it notes and pens may be needed to capture discussions and facilitate communication with aphasic individuals. Flipchart paper, pens.
Procedures	Participants are contacted 2 days before each session to check if they are attending. The protocol content was design using the core Bridges principles (see the ‘Core concepts of the intervention’ section). The content aims to incorporate these principles over the 4 weeks. Outcome measures are completed at the end of the fourth session.
Who provided	Each group should have the same three facilitators for each session. One stroke survivor, who will provide empathy and insight as they have experienced a stroke themselves. One speech and language therapist trained in Bridges who will provide self-management support and expertise in communication difficulties such as aphasia. Finally, one facilitator trained in Bridges providing self-management support. In this case, the latter is a PhD student and trainee health psychologist. Bridges training is one full day followed by a half day a few months later. The first day aims to help individuals build, evaluate and sustain a self-management approach with a focus on person-centred care. The second day allows individuals to reflect on their new practices, enabling the sharing of ideas and collective problem solving, as well as refreshing individuals on the core principles.
How (delivery)	Face-to-face in a group setting.
Where	The intervention will be run in community venues that are as convenient for each group member as possible.
When and how much	A four-part intervention running once a week for 4 weeks. Each session lasts 2 h and includes a break in the middle.
Tailoring	The intervention is tailored to the individual. For example, through individual goal setting and decision making. In addition, discussion topics are participant led ensuring they are relevant to those involved.
Modifications	Although there are clear time frames and content listed in the session plans, they are meant as a guideline and thus should be used flexibly in practice. For example, the order of events may vary and some aspects may be adapted to fit different contexts.
How well (fidelity)	Observations of the intervention will be carried out which will be cross referenced to the core principles of Bridges *and the original session plan*. This will enable an assessment of fidelity to the intervention protocol.

#### Group size

There is differing literature on suggested group size. The Chronic Disease Self-Management Program (CDSMP) has 10 to 15 [28] participants in each group whereas psycho education groups suggesting five to ten [29]. For this group self-management intervention, the group size will be conservative, five to eight stroke survivors. This accounts for the complex nature of stroke. For example, those with aphasia may require the use of additional communication techniques. Family and friends are welcome to attend the sessions.

#### Core concepts of the intervention

Social Cognition Theory (SCT) describes the mechanisms which may impact the likelihood of an individual engaging in new behaviours and suggest one of these is social support [30]. SCT suggests that social support along with knowledge and barriers feed directly into an individual’s self-efficacy (an individual’s belief in their own ability to complete a specific behaviour), which in turn contributes to goal setting and ultimately behaviour. This forms an iterative loop with behavioural outcomes feeding back into an individual’s self-efficacy. Consequently, self-efficacy is the central concept of self-management interventions and accordingly self-management interventions showing the most promise are based on self-efficacy [14].

Nine strategies used within Bridges will be translated into a group setting: reflection, knowledge, decision making, problem solving, goal setting, taking action, using resources, collaboration and self-discovery. As with the components of SCT, the Bridges strategies link together and can be iterative in nature. For example, an individual’s goal may be to walk more (setting goals) as they do so less than they used to (reflection), someone else may suggest walking a dog may make the task more enjoyable and recommend a charity that needs dog walkers (collaboration, knowledge, problem solving, using resources). The individual may then decide to go to the charity to sort this out (taking action, decision making). The outcome of this will then inform future decisions (reflection, mastery, taking action, goal setting).

#### Adverse events

There have been no adverse events reported from previous research on the Bridges one-to-one intervention or other stroke-specific group self-management interventions reported in the literature; hence, this study is considered minimal risk. However, the population we are studying has a natural disease progression, and severe events such as a death may occur. In addition, the research is at the feasibility stage so unexpected risks may occur. As a result, any adverse events will be documented and the ethics committee notified. If any patients present with clinically relevant scores, for example if the Hospital Anxiety and Depression Scale shows severe anxiety and/or depression, we will notify their GP (with their consent).

### Waitlist group

The waitlist control group will not receive any interaction with the research team during the 6-month wait period. Participants in this condition will complete baseline measures at the start and the end of the waitlist period before randomisation as well as completing questions about demographics as specified above. They will continue to engage with usual care as they would if not taking part in research. Once the wait period is completed, they will take part in the intervention. Outcome measures for this group will be completed at the end of the intervention and again at 2 weeks post intervention. For both the intervention and the waitlist group, questions 2 (what will the level of adherence and attendance to the group self-management intervention be?) and 3 (what will follow-up response rates to questionnaires be?) will be answered using documentation of attendance to the intervention and the return rate of follow-up questionnaires. Participants may withdraw from the waitlist group at any time if they request to do so.

### Management

#### Study management

The study is overseen by a seven-person multidisciplinary management panel who meet to discuss progress and any issues that may arise. This includes input from medical, psychological, statistical, and physiotherapy backgrounds. In addition, this panel includes a stroke survivor who has been involved with the study from its conception. This includes the study design as well as the development of study documents, ensuring both are accessible and acceptable to the target population.

#### Data management

Where possible data entry will be electronic via electronic devices. This will occur through the programme ‘RedCap’. All data transfer and storage will be in line with the Data Protection Act (1998). Data entered manually will be subject to a yearly audit by the trial manager (data checking against raw scores). Identifiable information collected manually will be stored securely and kept for 10 years by University College London. A collection log will be in place to ensure all data is accounted for, and to act as a prompt for follow-up data collection. All study data will be stored and analysed in the UK.

### Evaluation

#### Outcome measures

As this is a feasibility study, no primary outcome was identified in accordance with guidelines [19]. Four quantitative measures and two qualitative processes will be used in this study as detailed below.

#### Interviews

It is vital that any complex intervention is acceptable to the target population [19]. Question 5 (how acceptable to stroke survivors is it to deliver Bridges in a group setting?) will be answered through interviews conducted with stroke survivors who have attended the group self-management intervention. Purposeful sampling will enable a range of demographics to be included. A sample size is not specified as, in keeping with guidelines for qualitative research, the process of recruitment and analysis are iterative, continuing until no new themes emerge from the data. Semi-structured interviews will be conducted as they allow flexibility in the ordering of questions and are widely used in health research [31]. All interviews will be conducted post intervention by a researcher who has not been involved with the project beforehand to minimise potential observer effects. The interviews will take place where the stroke survivor feels most comfortable; it is thought the majority of interviews will be in their own homes. The benefit of this is that being non-clinical, it may emphasise that the interviews had no impact on clinical care and again encourage more honest answers. Participants will have the option to bring a friend or family member with them. Each interview will be recorded using a dictaphone.

The topic guide has been developed by two researchers (EC and FJ). The topic guide was developed drawing on guidelines for interviews in health research to answer key questions relevant to the feasibility of the intervention [32]. The topic guide comprises four parts: (1) learning about the individual’s stroke journey, (2) experiences of taking part in the group self-management intervention, (3) life after the group self-management intervention and (4) questions about the research methodology (see Additional file 1).

#### Focus group

Question 6 (how acceptable is it to family/friends/carers to deliver Bridges in a group setting?) will be explored using focus groups. An individual’s social support can influence their likelihood to engage in new behaviours [30]. It is therefore important to understand the experiences of the friends, family and carers that attended the group self-management interventions as they are likely to be part of the self-management journey. A maximum of 12 family/friends/carers will be recruited to take part in up to two post intervention focus group/s, each with four to eight participants in. This is a balance between giving each member the space to voice opinion and ensuring enough data is captured [33]. Family/friends/carers who attended the group self-management intervention will be invited to a focus group to give feedback on the intervention. Focus groups have numerous advantages including stimulating discussion [33] and the ability to probe for more detail when potentially interesting topics arise [34].

Focus groups will take place in a non-clinical venue as this will minimise the possibility that individuals feel that what they say may impact their clinical support. Each focus group will last around 60 min and be audio recorded. The room will be set up in accordance with published recommendations, for example, a round table setting conducive to discussion [33]. The topic guide will be divided up into three parts: Firstly, attendees will be asked about their experiences of the group in general; secondly, about limitations, challenges and possible benefits, as well as what they may have learnt from the group and in turn what they feel the person they attended with may have learnt from the group. The discussion will end with questions asking about future possibilities for group self-management interventions and exploring whether the intervention has any longevity. The questions that will be asked during each focus group were developed with a stakeholder group using questions that utilise everyday language. For example, do you feel confident that you and the stroke survivor you attended with can carry on under your own steam? (see Additional file 2).

#### Quantitative outcome measures

Question 7 (what is the magnitude and variability of change in outcome measures post intervention?) will be answered using quantitate outcome measures. MRC guidelines that state identifying a single primary outcome in complex interventions may not make the best use of data [23]. Identifying a range of measures (both qualitative and quantitative) will maximise the chance that unexpected consequences can be captured.

The Stroke and aphasia quality of life measure-39 (SAQOL) uses a 39 item scale which is split into four subdomains (physical, psychosocial, communication and energy) to assess quality of life [35]. The Stroke self-efficacy scale (SSES) gives an indication of an individual’s belief in their own ability to complete certain tasks [36]. The Nottingham extended activities of daily living scale (NEADLS) will be used to get insight into the day-to-day functioning of each individual [37]. The Hospital Anxiety and Depression Scale (HADS) will give insight into an individual’s mood [38]. Participants are given the option to complete questionnaires over the phone or in person with a member of the research team. If posted participants are called to confirm if they have received the questions and to see if they require any assistance.

### Analysis

Analysis of data will be completed by the research team which includes a statistician and an expert in qualitative research. Due to the nature of the intervention and extended follow-up periods for the intervention group, it is not possible to blind participants or those analysing data to the condition. If missing data occur, we will describe their numerical impact on the actual sample observed as well as the possible reasons for dropout, which we aim to collect as carefully as possible. Given the nature of the study and the limited sample size, we do not plan on doing formal and complex modelling (e.g. multiple imputation). However, we will assess the impact of possible missingness mechanisms on the results of the data analysis.

#### Questions 1–4

Questions 1 to 4 (1. How many stroke survivors are eligible and how quickly can they be recruited? 2. What will the level of adherence and attendance be? 3. What will follow-up response rates to questionnaires be? 4. Are participants willing to be randomised to a waitlist control in a group self-management study?) will be answered using frequency counts and descriptive statistics. Session attendance will be monitored to provide dropout and partial-completion rates. Those who do not complete the intervention will be asked why and the answers categorised to determine the most common reasons. To gain insight into recruitment, the proportion of stroke survivors who met the eligibility criteria will be documented as well as how many were approached to take part and the length of time taken to recruit all 60. Willingness of participants to be randomised will be assessed using the number declining to participate as a result of randomisation will be documented. To assess follow-up rates, the date which questionnaires are sent out and the date returned will be recorded to give an average response time. The number of non-returns will also be documented.

#### Question 5 and 6

Questions 5 (how acceptable to stroke survivors is it to deliver Bridges in a group setting?) and 6 (how acceptable is it to family/friends/carers to deliver Bridges in a group setting?) will be answered using qualitative interviews (stroke participants) and focus groups (family and friends). Interviews will be transcribed and analysed by two researchers using inductive thematic analysis. Inductive thematic analysis is recommended for preliminary health service research and when key themes reflecting variations in the data need to be identified as with this project [29, 30]. Codes will be created using phrases or words drawn directly from the data. These will then be grouped to develop categories and themes. Both researchers will write a summary of their interpretations and discuss them together, including codes and their descriptive groupings. Any disagreement will be discussed by the researchers and at subsequent management meetings if further resolution is needed. Thematic analysis will then involve iteratively exploring which themes are identified across the data set. The data set will then be re-read to find illustrative examples of themes and adjusting them to reflect any new data gathered [39]. Data will be managed using Nvivo 10.

#### Question 7

Question 7 (What is the magnitude and variability of change in outcome measures post intervention?) will be answered using an analysis of covariance (ANCOVA). In line with guidelines [19], there is no formal power calculation for the study, meaning results are not statistically powered to find an effect. Therefore, results are to be considered only as preliminary. An ANCOVA will analyse the results comparing pre-post intervention scores (within subjects) and the intervention to the waitlist group (between subjects). Demographics will be included as co-variates where appropriate, for example living situation and time post stroke. Variability in outcome measures will provide the information needed to calculate the power for future research on this topic.

### Dissemination

All co-applicants will facilitate dissemination to a range of sources including academics, stroke survivors and the relevant funders, e.g. National Institute for Health Research—Research for patient benefit. If feasible, dissemination will include progression to the subsequent randomised control trial. In addition, dissemination will include a mixture of presentations, publications and web-based social media (e.g. twitter).

## Discussion

Interest is growing in group self-management interventions as they may facilitate the long-term management of stroke, an area which is currently understudied. However, before group self-management interventions can be evaluated as part of a fully powered trial, the feasibility of such interventions need to be assessed. This protocol detailed the methodology and methods that will be used for a funded exploration into whether it is feasible to deliver an existing self-management intervention (Bridges) in a group setting.

When designing the study, care was taken to use methodology and methods advocated by current research guidelines. This includes the Medical Research Guidelines for complex interventions [21], TiDieR guidelines for intervention descriptions [22], SPIRIT reporting guidelines [20], and NIHR advice on feasibility studies [19]. The study also has a multidisciplinary management panel who co-designed the methodology, including a stroke survivor. However, there are some limitations of the proposed study that should be addressed. The recruitment strategy means that participants will be recruited from one hospital which limits the geographical reach. Although it was decided that this was acceptable for the current study as the priority is to assess feasibility, a larger future trial should include more than one recruitment site to increase the generalisability of the data. In addition, as stated in MRC guidelines, it can be problematic for complex interventions to be fully standardised, especially as group self-management interventions should retain enough flexibility to adapt to context and ensure they are tailored to each individual [11]. This makes replicability and fidelity to protocol harder to asses. To overcome this, the TiDieR guidelines were used which provide a reporting procedure for interventions that is flexible enough to incorporate adaptions to context [22].

Once completed, this research will determine if the intervention should progress to a full RCT. It will do so by exploring the acceptability of the intervention, the occurrence of serious adverse events occur, the appropriateness of research measures and adherence rates which should be over 85%. It will also inform the design of future work assessing the efficacy of a group self-management intervention. It will do so in a number of ways. Firstly, this research will help determine the project timeline as information about the number and rate of eligible participants that can be recruited as well as the follow-up questionnaire return rate will be available. Secondly, it will determine whether future work should randomise participants to a condition as their willingness to do so will be documented. Thirdly, the design of the group self-management intervention may be adapted based on the acceptability to stroke survivors and family/friends/carers. Finally, the information to conduct a power calculation which will determine the sample size of a larger trial will be available as a result of documented dropout rates and standard deviation of outcome measures.

## Additional files

Additional file 1: Interview Topic Guide. (DOCX 23 kb)

Additional file 2: Focus Group Topic Guide. (DOCX 16 kb)

## Abbreviations

ANCOVA: Analysis of covariance
CDSMP: Chronic Disease Self-management Program
GUSTO: GroUp Self-management intervention for sTrOke
HADS: Hospital Anxiety and Depression Scale
HASU: Hyper Acute Stroke Unit
NEADL: Nottingham extended activities of daily living scale
NIHR: National Institute for Health Research
PTC: Permission To Contact register
SAQOL: The Stroke and aphasia quality of life measure-39
SCT: Social Cognition Theory
SPIRIT: Standard Protocol Items: Recommendations for Interventional Trials
SSES: Stroke self-efficacy scale
TIDieR: Template for Intervention Description and Replication
UK: United Kingdom

## References

[1] Stroke Association. State of the Nation: Stroke statistics 2017 [Internet]. 2017. Available from: https://www.stroke.org.uk/sites/default/files/state_of_the_nation_2017_final_1.pdf. Accessed 4 Feb 2017.

[2] McKevitt C. A national survey to investigate the long-term unmet needs of UK stroke survivors. Fudge N., Crichton S., Sheldenkar a, Rudd a, Wolfe CDA, Rothwel PM, et al., editors. Int J Stroke [Internet]. 2010;5. Available from: 10.1111/j.1747-4949.2010.00515.x. Cited 9 Aug 2013.

[3] O’Neill D, Horgan F, Hickey A, McGee H (2008). Stroke is a chronic disease with acute events. BMJ.

[4] Ellis-Hill C. Going home to get on with life: patients and carers experiences of being discharged from hospital following a stroke. Robison J, wiles R, McPherson K, Hyndman D, Ashburn a, editors. Disabil Rehabil. 2009;31(2):61. 10.1080/09638280701775289 19152154

[5] White J, Dickson A, Magin P, Tapley A, Attia J, Sturm J (2014). Exploring the experience of psychological morbidity and service access in community dwelling stroke survivors: a follow-up study. Disabil Rehabil.

[6] Coulter A, Roberts S, Dixon A. Delivering better services for people with long-term conditions: building the house of care [internet]: Kings Fund; 2013. Available from: https://www.kingsfund.org.uk/sites/files/kf/field/field_publication_file/delivering-better-services-for-people-with-long-term-conditions.pdf. Accessed 12 Feb 2017.

[7] Sadler E, Wolfr C, Jones F, McKevitt C. Exploring stroke survivors’ and physiotherapists’ views of self-management after stroke: a qualitative study in the UK. PubMed J [Internet]. 2017. Available from: https://ncbi.nlm.nih.gov/labs/articles/28283483/. Cited 10 Apr 2017. 10.1136/bmjopen-2016-011631 PMC5353340 28283483

[8] Care Quality Commission. Supporting life after stroke: a review of services for people who have had a stroke and their carers. Care Quality Commission [Internet]; 2011; Available from: https://www.cqc.org.uk/sites/default/files/documents/supporting_life_after_stroke_national_report.pdf. Acessed 1 Mar 2017.

[9] Intercollegiate Stroke Working Party. National Clinical Guidelines for stroke. Royal College of Physicians [Internet]; 2012; Available from: http://www.rcplondon.ac.uk/sites/default/files/national-clinical-guidelines-for-stroke-fourth-edition.pdf. Accessed 4 Feb 2017.

[10] Jones F, Poestges H, Brimicombe L (2016). Building bridges between healthcare professionals, patients and families: a coproduced and integrated approach to self-management support in stroke. NeuroRehabilitation.

[11] Bandura A. Self-efficacy: the exercise of control. Unites States: W. H. Freeman; 1977.

[12] Lorig KR, Sobel DS, Stewart AL, Brown BWJ, Bandura A, Ritter P, et al. Evidence suggesting that a chronic disease self-management program can improve health status while reducing hospitalization. Med Care. 1999;37:5–14. 10.1097/00005650-199901000-00003 10413387

[13] Jones F. Strategies to enhance chronic disease self-management: how can we apply this to stroke? Disabil Rehabil. 28(13–14):841. 10.1080/09638280500534952 16777771

[14] Fryer C.E., Luker J.A., Mcdonnell M.N., Hillier S.L. Self management programmes for quality of life in people with stroke [internet]. Cochrane Database Syst Rev. 2016. Available from: http://as.wiley.com/WileyCDA/Brand/id-6.html. Accessed 4 Feb 2017. 10.1002/14651858.CD010442.pub2 PMC6450423 27545611

[15] Bridges Self Management [Internet]. Bridges. Available from: http://www.bridgesselfmanagement.org.uk/. Cited 10 Mar 2016.

[16] Vassilev I, Rogers A, Kennedy A, Koetsenruijter J (2014). The influence of social networks on self-management support: a metasynthesis. BMC Public Health.

[17] Morris RL, Kennedy A, Sanders C. Evolving “self”-management: exploring the role of social network typologies on individual long-term condition management. Health Expect. 2015;19:1044–61. 10.1111/hex.12394 PMC5053258 26284341

[18] Catalano T, Dickson P, Kendall E, Kuipers P, Posner TN (2003). The perceived benefits of the chronic disease self-management program among participants with stroke: a qualitative study. Aust J Prim Health.

[19] National Institute for Health Research. Feasibility an pilot studies: NIHR; 2013.

[20] Publications & downloads – SPIRIT Statement [Internet]. Available from: http://www.spirit-statement.org/publications-downloads/. Cited 25 Jan 2017.

[21] Craig P, Dieppe P, Mcintyre S, Nazareth I, Mitchie S, Petticrew M (2011). Developing and evaluating complex interventions: the new Medical Research Council guidance. BMJ.

[22] Better reporting of interventions: template for intervention description and replication (TIDieR) checklist and guide | The EQUATOR Network [Internet]. Available from: http://www.equator-network.org/reporting-guidelines/tidier/. Cited 9 Dec 2016.

[23] Medical Research Council. Developing and evaluating complex interventions: new guidance [Internet]. 2006. Available from: http://www.mrc.ac.uk/documents/pdf/complex-interventions-guidance/. Cited 22 Jan 2015.

[24] Julious S (2005). The size of a pilot study for a clinical trial should be calculated in relation to considerations of precision and efficiency. Pharm Stat.

[25] Sim J, Lewis M (2012). The size of a pilot study for a clinical trial should be calculated in relation to considerations of precision and efficiency. Clin Epidemiol.

[26] Jones F, Mandy A, Partridge C. Changing self-efficacy in individuals following a first time stroke: preliminary study of a novel self-management intervention. Clin Rehabil. 2009;23:522–33. 10.1177/0269215508101749 19403556

[27] Clark E, Bennett K, Ward N, Jones F (2016). One size does not fit all—stroke survivor’s views on group self-management interventions. Disabil Rehabil.

[28] Lorig KR, Sobel DS, Stewart AL, Brown BW, Bandura A, Ritter P (1999). Evidence suggesting that a chronic disease self-management program can improve health status while reducing hospitalization: a randomized trial. Med Care.

[29] Brown NW, Edd L. Psychoeducational groups: process and practice. New York: Taylor & Francis; 2011. p. 284.

[30] Bandura A (1986). Social foundations of thought and action: a social cognitive theory.

[31] Jamshed S (2014). Qualitative research method-interviewing and observation. J Basic Clin Pharm.

[32] Green J, Throrogood N. Qualitative methods for health research. 3rd ed. London: SAGE Publications; 2014.

[33] Wong LP (2008). Focus group discussion: a tool for health and medical research. Singap Med J.

[34] Morgan DL. Focus groups as qualitative research. California: SAGE; 1997. p. 92.

[35] Hilari K. Psychometric properties of the Stroke and Aphasia Quality of Life Scale (SAQOL-39) in a generic stroke population. Lamping DL, smith SC, Northcott S, lamb a, Marshall J, editors. Clin Rehabil 2009;23(6):544. 10.1177/0269215508101729 19447841

[36] Jones F, Partridge C, Reid F (2008). The Stroke Self-Efficacy Questionnaire: measuring individual confidence in functional performance after stroke. J Clin Nurs.

[37] Sarker S-J, Rudd AG, Douiri A, Wolfe CDA (2012). Comparison of 2 extended activities of daily living scales with the Barthel Index and predictors of their outcomes: cohort study within the South London Stroke Register (SLSR). Stroke J Cereb Circ.

[38] Aben I, Verhey F, Lousberg R, Lodder J, Honig A (2002). Validity of the beck depression inventory, hospital anxiety and depression scale, SCL-90, and hamilton depression rating scale as screening instruments for depression in stroke patients. Psychosomatics.

[39] Braun V, Clarke V (2013). Successful qualitative research: a practical guide for beginners.

