# Supplementary material for: Research protocol: investigating the feasibility of a group self-management intervention for stroke (the GUSTO study)
Source: Pilot Feasibility Stud. 2018 Jan 11;4:31. doi: 10.1186/s40814-017-0220-1 (PMC5765599; doi:10.1186/s40814-017-0220-1)
Supplement: Supplementary file 1 — Interview Topic Guide. (DOCX 23 kb) [file 40814_2017_220_MOESM1_ESM.docx]

Interviews transcript (Version 1 - 11/10/2016)

Note: These questions may be asked in different orders depending on the flow of the interview.

**Start**

*Interviewer introduces themselves and starts with rapport building questions such as, job, where they live, family.*

**Your story**

- *Can you tell me about your stroke/ your experience of stroke*
- *What support have you said outside the hospital?*

**Group self-management**

- Where were you in your stroke journey when you took part in the group?
- Could the group have been delivered at a different time in your stroke journey?
- Have you learnt anything about yourself during the group?
- How did the group teach you this?
- Have you learnt anything from others in the group?
- Do you think others in the group learnt anything from you?
- How have you felt supported by / supported other group members?

**Post group / future**

- Are you doing anything differently since the group?
- If you were having difficulty with something, what would you now?
- Is that different to what you would have done before the group?
- Is there anything that you will take from the group into your future?
- How confident do you feel to carry on under your own steam?

**Research**

- How did you feel being randomised to condition (can explain to participant if needed)?
- Do you have any feedback about research?

**Closing question**

- If you had two wishes for the groups what would they be?

**End**

**Examples of phrases used to prompt participants into expanding on a point:**

- Can you tell me more about that?
- Could you expand on that?
- What makes you say that?
- How do you feel about that?
- How did that make you feel?
- Why do you think that was?
- How did that situation arise?
- How was that issue resolved?
- What were the implications of/for that?
- How did that change over time?
- Could you explain further?
