# Supplementary material for: Research protocol: investigating the feasibility of a group self-management intervention for stroke (the GUSTO study)
Source: Pilot Feasibility Stud. 2018 Jan 11;4:31. doi: 10.1186/s40814-017-0220-1 (PMC5765599; doi:10.1186/s40814-017-0220-1)
Supplement: Supplementary file 2 — Focus Group Topic Guide. (DOCX 16 kb) [file 40814_2017_220_MOESM2_ESM.docx]

# Topic Guide for Focus-Group: 19/14/2017

## Introduction: 10 min

- Intro (Katie) + Housekeeping (Ella)

*Icebreaker: All attendees introducing themselves to the group using postcards + explaining what they hope to get out of the meeting today*

## Discovery: 30 min

*Participants interviewing each other/ fill in form individually (1.-3.) for 15 minutes.*

### Think of the Bridges group sessions.

- Can you describe your **absolute highlight** of the sessions you attended please?
- Did the group **inspire you** or the person you attended the sessions with **to try out something new**? If yes, can you tell me your story about doing something different during or after the sessions please? Do you feel confident that you and the stroke survivor you attended with can carry on under your own steam? If not, what could have been done differently to inspire you or the person you attended with?

### Now think of a special time when you met a person or a group of people who had a profound effect on your life. That person or group might have believed in you, supported or inspired you.

- Who was it?
- What made the encounter with this person or group so special?
- How is your life different because of meeting this person or group?

### Think of the Bridges group sessions again. If you had three wishes for future groups - what would they be?

*Participants summarise and present the answers from their interview partners to the whole group. Facilitated group discussion follows. (15 minutes)*

- Possible prompts: thoughts about facilitators, hearing from others, timing, impact on their confidence and behaviour

## Dream: 5-10 min

### In an ideal world, what would a perfect group session be like that is a life changing experience for you and the stroke survivor you are attending with?

*Facilitated group discussion.*

## Design: 5-10 min

### What needs to happen to ensure that every single group session has a profound effect on your and the stroke survivor’s life?

*Facilitated group discussion + writing down provocative propositions/shared ideals (in present tense)*

## Destiny and Closing: 5 min

### What are the first next steps to make this happen?
